# Environmental noise and sleep and mental health outcomes in a nationally representative sample of urban US adolescents

**DOI:** 10.1097/EE9.0000000000000056

**Published:** 2019-06-25

**Authors:** Kara E. Rudolph, Aaron Shev, Diana Paksarian, Kathleen R. Merikangas, Daniel J. Mennitt, Peter James, Joan A. Casey

**Affiliations:** a Department of Emergency Medicine, School of Medicine, University of California, Davis, Sacramento, California; b Genetic Epidemiology Research Branch, National Institute of Mental Health, Bethesda, Maryland; c Department of Electrical and Computer Engineering, Colorado State University, Fort Collins, Colorado; d Department of Population Medicine, Harvard Medical School and Harvard Pilgrim Health Care Institute, Boston, Massachusetts; e Division of Environmental Health Sciences, School of Public Health, University of California, Berkeley, California

**Keywords:** Adolescent, Anxiety disorders, Conduct disorder, Depressive disorder, Major, Mental health, Noise, Substance-related disorders, United States Environmental Protection Agency

## Abstract

**Background:**

Environmental noise has been linked to negative health outcomes, like poor sleep, poor mental health, and cardiovascular disease, and likely accounts for more than 1 million disability-adjusted life years annually in Western Europe. Adolescence may be a particularly sensitive period for noise exposure due to an increased need for sleep, failure to meet sleep guidelines, and increased risk for first onset of some mental health disorders. However, the potential health effects of living in high-noise environments have not been studied in US adolescents, rarely in European adolescents, and mental health outcomes studied have not corresponded to diagnoses from the Diagnostic and Statistical Manual of Mental Disorders (DSM).

**Methods:**

Using a US-based nationally representative survey of urban adolescents (N = 4,508), we estimated associations of day-night average sound levels exceeding the US Environmental Protection Agency’s 55 decibel limit with sleep outcomes and lifetime mental health DSM diagnoses. We implemented doubly robust targeted minimum loss-based estimation coupled with propensity score matching to account for numerous potential adolescent, household, and environmental confounders.

**Results:**

Living in a high- versus low-noise Census block group was associated with later bedtimes on weeknights (0.48 hours, 95% confidence interval [CI] = –0.15, 1.12) and weekend nights (0.65 hours, 95% CI = 0.37, 0.93), but not with total hours slept. Associations between living in a high- versus low-noise Census block group and mental disorders were mixed, with wide CIs, and not robust to sensitivity analyses.

**Conclusions:**

We find evidence for an association between residence in a high-noise area and later bedtimes among urban adolescents but no consistent evidence of such an association with mental health disorders.

## Abstract 2

Supplemental Digital Content is available in the text.

**What this study adds**

This is the first US study to examine a relation between environmental noise and adolescent health. We identified communities where day-night average sound levels exceeded the US Environmental Protection Agency’s limit of 55 decibels (which we call high-noise). We then estimated associations between noise and sleep and mental health diagnoses from the Diagnostic and Statistical Manual of Mental Disorders, Fourth Edition (DSM-IV) among a nationally representative sample of US urban adolescents. We used machine learning and doubly robust estimation to control for numerous potentially confounding variables at the community, family, and individual levels. We found that residence in a high-noise communities was associated with later bedtimes but not with mental health.

## Introduction

Environmental noise—unwanted or extraneous sound from sources like traffic and airports—has been linked to negative health outcomes including sleep disturbances, poor mental health, and cardiovascular disease.^1^ Among adults, a pooled analysis of polysomnographic and self-reported awakenings studies found consistent evidence that nighttime noise caused cortical awakenings and sleep disturbances.^2^ Nighttime noise may also affect sleep duration, efficiency, and insomnia.^2,3^ These sleep disturbances may harm mental health,^4,5^ but depression and anxiety may also arise independently from sustained central autonomic arousal due to chronic noise exposure.^6,7^ Considering these negative health effects together, exposure to high environmental noise levels is estimated to result in the loss of at least 1 million disability-adjusted life years annually in Western Europe.^8^

Adolescents may be particularly sensitive to negative health effects from environmental noise due to their increased need for sleep^9^ coupled with failure to meet sleep guidelines,^10^ and risk of mental disorder onset during this developmental period.^11^ However, despite adolescence being a potentially developmentally vulnerable period, there is little research on the health effects of noise exposure in this subgroup.^12,13^ In terms of sleep outcomes, three studies among children and early adolescents reported associations between noise and reduced sleep quality^14^ and greater sleep problems.^15,16^ In terms of mental health outcomes, studies among children and adolescents generally found no association between noise and depression and anxiety scales,^13,17^ but generally did find associations between noise and inattention and hyperactivity.^15,16,18–20^ However, none of these studies examined mental health outcomes that corresponded with diagnostic criteria. Sympathetic nervous system activation, noise annoyance, social cohesion, and physical activity have been proposed as mediators and/or moderators of the possible relation between noise exposure and mental health.^21,22^

If a relation exists between environmental noise and adolescent sleep and mental health, there are reasons to believe it would be strongest in urban areas. First, the prevalence and incidence of mental disorders such as schizophrenia^23^ and depression and anxiety^24,25^ tend to be greater in urban areas. Short sleep duration is also more common among adults living in more urban locations.^26^ Exposure to environmental noise varies spatially in type and magnitude and is greater in urban areas.^27^ Finally, previous research demonstrated that the strength of association between adolescent depression and anxiety and another contextual variable—neighborhood deprivation—varied by level of urbanicity, with the strongest relation in urban areas.^28^

Our objectives were two-fold. First, we identified US communities that exceed the US Environmental Protection Agency’s (EPA) day-night average sound level (Ldn) of <55 decibels (dB)^29^ using a high-resolution nationwide noise model. These recommended levels are likely exceeded in many communities,^30^ but no recent estimates of noise exceedances in the US exist. Second, we tested the policy-relevant hypothesis that living in communities where environmental noise exceeds the US EPA threshold is associated with worse sleep and higher prevalence of mental health disorders corresponding to the DSM-IV in a nationally representative sample of US urban adolescents.

## Methods

We examined this research question in an urban subsample of the National Comorbidity Survey Replication Adolescent Supplement (NCS-A), a nationally representative survey of adolescents living in the contiguous US conducted 2001–2004 (N = 4,508 urban adolescents). Details of the sampling design and procedures have been published previously.^31–33^ Briefly, household and school samples of 13–18 year-olds participated in face-to-face interviews, with an overall response rate of 75.6%. Parents provided written informed consent (except for emancipated minors) and adolescents provided assent. Study procedures were approved by the human subjects committees of Harvard Medical School and the University of Michigan. This analysis, utilizing de-identified data, was determined nonhuman subjects research by the University of California, Berkeley, and University of California, Davis.

### Outcomes

We examined several sleep and mental health outcomes. Sleep patterns included the following: (1) typical hours slept per weeknight; (2) per weekend night; (3) typical bedtime on a weeknight; and (4) weekend night, based on adolescent response to open-ended questions. Mental disorders included the following: (1) lifetime anxiety or depressive disorder (major depressive disorder, dysthymia, or any anxiety disorder) based on adolescent interview in accordance with prior recommendations^34^; (2) lifetime behavioral disorder (conduct disorder or oppositional defiant disorder) based on adolescent or caregiver interviews combined at the symptom level, among those with caregiver interviews; (3) lifetime attention-deficit/hyperactivity disorder (ADHD) with impairment based on caregiver interviews; and (4) lifetime substance use disorder based on adolescent interview. Disorders were categorized using adolescent and/or caregiver endorsement of symptoms, as specified above, as has been done previously,^35^ using a modified version of the World Health Organization Composite International Diagnostic Interview (CIDI) Version 3.0, administered by trained interviewers. The resulting disorder categorizations correspond to DSM-IV diagnoses according to a blinded clinical reappraisal substudy.^36^ Caregiver reports of adolescent behavioral symptoms were ascertained by a self-administered questionnaire completed by caregivers of 2,645 (59%) urban adolescents (as well as 2,242 suburban and 1,596 rural adolescents).

### Sample

We examined associations among adolescents living in urban centers for our primary analysis (N = 4,508), and include adolescents living in urban fringe areas (N = 3,304) and nonurban areas (N = 2,311) in the Supplement. Urban areas were defined as central counties within standard metropolitan statistical areas (MSAs) and having populations ≥ 100,000. Urban fringe and nonurban areas, which we consider in the Supplement, were defined as noncentral counties within MSAs or those < 100,000 and as small-large towns outside of MSAs or rural areas.

### Environmental and sociodemographic measures

Our exposure of interest was ambient outdoor noise over the US EPA threshold of 55 dB. Ambient outdoor noise was defined by Ldn, the sound level averaged over the year and A-weighted to mimic the sensitivity of the human auditory system.^1^ Ldn is calculated within a 24-hour period with 10 dB added to levels between the hours of 10 pm and 7 am. The additional 10 dB upweights nighttime noise, reflecting increased sensitivity to noise at night. The metrics were projected to a 270 m × 270 m grid across the contiguous US from geospatial sound models, models derived from over 1.5 million hours of acoustical data (sampled 2000–2014) and dozens of geospatial features accounting for both natural and anthropogenic sources.^27,37^ Ldn was averaged within each US Census block group. For the primary analyses, noise was dichotomized as high (≥55 dB) versus low (*<*55 dB). The sensitivity analyses described below used alternative exposure definitions.

We included numerous potentially confounding variables in our models, including aspects of the environment: air pollution (modeled block group level nitrogen dioxide [NO_2_] in 2000),^38^ satellite-based greenness (block group level normalized difference vegetative index [NDVI] in 2000),^39^ average high temperature (county-level in 2000),^40^ population density, and neighborhood socioeconomic status (block group level in 2000, measured as a 6-item index from the US Census that includes median household income, median housing value, occupation types, education levels, and sources of wealth)^41^; adolescent characteristics: sex, age, race/ethnicity (black, white, Hispanic/Latino, other), English as primary language, citizenship status, immigrant generation, region of the country (Northeast, Midwest, South, West), religion (protestant, catholic, other religion, no religion), whether the adolescent lived her/his whole life with his/her mother and/or father, student status; and family characteristics: family income (log-transformed), maternal age at birth of the adolescent, maternal education, parental marital status, and family conflict tactics (presence of psychological aggression, moderate forms of physical assault, and severe forms of physical assault separately for adolescent-parent dyad and parent-parent dyad, as have been included in previous NCS-A analyses relating contextual exposures to mental health).^28,42^

### Statistical approach

We first imputed missing values using multiple imputation by chained equations, which assumes the data are missing at random conditional on the variables in the imputation model,^43^ producing 30 imputed datasets. Out of the 36 variables used for the analysis, 19 variables were missing between 0.03% and 43% of observations. Parent marital status and maternal level of education had highest levels of missingness at 43% and 30%, respectively. The remaining variables had less than 4% missing. An analysis that ignores the missingness assumes that the data are missing completely at random—that is, missingness is independent of both observed and unobserved variables.^44^ This is a strong and typically unrealistic assumption.^44^ Addressing missingness through multiple imputation instead assumes that the data are missing at random—that is, missingness can depend on observed variables that are included in the missingness model.^44^ We included all variables used in our analysis in all missingness models, which is necessary for congeniality,^45^ as well as many additional variables at the individual, family, and Census block group levels for a total of 122 variables and 40 additional second order interactions that improved model fit. We assessed the quality of the imputation by comparing densities of the imputed and observed values, as recommended.^43^ We completed analysis on each dataset and then pooled the results using combining rules of Rubin^46^.

We then used full matching on the propensity score^47^ for living in a high-noise area, including exact matching on sex and matching within narrow calipers on family income and neighborhood socioeconomic status, given the importance of these factors in adolescent mental health.^35,48^ Web Figure 1; http://links.lww.com/EE/A50 shows the resulting propensity score and covariate balance across the two exposure groups. The resulting frequency weights from the matching procedure were multiplied by the NCS-A sampling weights for analysis. We used a doubly robust substitution estimator, targeted minimum loss-based estimation,^49^ which incorporates the previously estimated propensity scores and includes for the aforementioned covariates in the outcome model, to estimate the adjusted association of exposure to high noise levels with each outcome. Confidence intervals (CIs) were further adjusted to account for multiple testing using a false discovery rate of 5%.^50^ Additional details of our approach are available in the Web Appendix 1; http://links.lww.com/EE/A50.

R Version 3.3.1 was used and code to replicate these analyses is provided: https://github.com/kararudolph/code-for-papers/blob/master/NCSAnoisepaper.R.

### Sensitivity analyses

We assessed the sensitivity of our results to extrapolations beyond the support of the data by limiting our analysis to the subsample of urban adolescents (N = 1,880) with counterparts in the opposite exposure group with similar propensities to live in high-noise areas (Web Appendix 2, Web Figure 2; http://links.lww.com/EE/A50).^51–53^ However, because such restriction results in no longer being able to interpret the results as applying to the nationally representative sample of urban US adolescents, we use the unrestricted sample for the primary analysis.

We also repeated our analyses using three alternative exposure definitions. First, we repeated our analyses using a continuous measure of noise. Second, we dichotomized noise at the US Federal Aviation Administrations Ldn threshold of 65 dB.^54^ Third, we used a data-driven approach to dichotomize community noise as high or low by using hierarchical clustering of several measures of block group level noise (Web Appendix 3; http://links.lww.com/EE/A50).^55^

## Results

**Figure 1.**
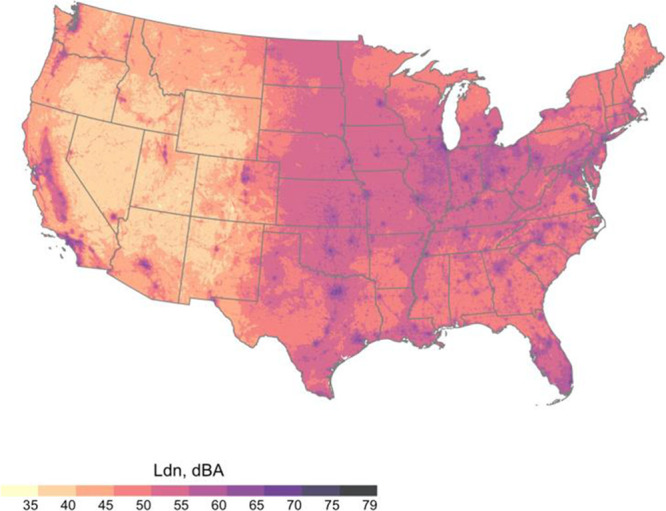
Ldn in dB across the continental United States, 2000–2014. Values estimated from a geospatial sound model.

**Figure 2.**
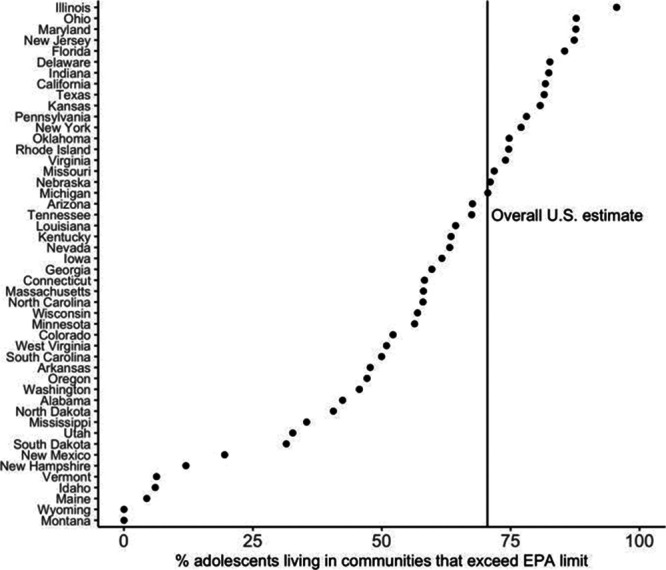
State-specific estimates of the percentage of adolescents living in block groups where average day-night noise exceeded 55 dB, 2000–2014. Data on adolescents were downloaded from the 2010 US Census.

Geospatial sound model projections in the period 2000–2014 revealed elevated noise levels in many US communities, particularly in those with dense populations and transportation networks (Figure 1). The 4,508 urban-dwelling adolescent NCS-A participants lived in 2,751 block groups with an average Ldn of 57.2 dB (SD = 4.6). Nearly 91% of participants lived in communities where Ldn exceeded 55 dB (i.e., high-noise). Non-Hispanic Black and Hispanic/Latino participants more often lived in high-noise communities (Table 1). In contrast, 75% of urban fringe adolescents and 18% of nonurban adolescents lived in communities where Ldn exceeded 55 dB (Web Table 1; http://links.lww.com/EE/A50). Overall, we estimated that nearly 71% of US adolescents (n = 29,698,782) lived in block groups where Ldn exceeded the US EPA limit of 55 dB. Percentages varied by state, from 0% in Montana and Wyoming to over 95% in Illinois (Figure 2).

**Table 1:**
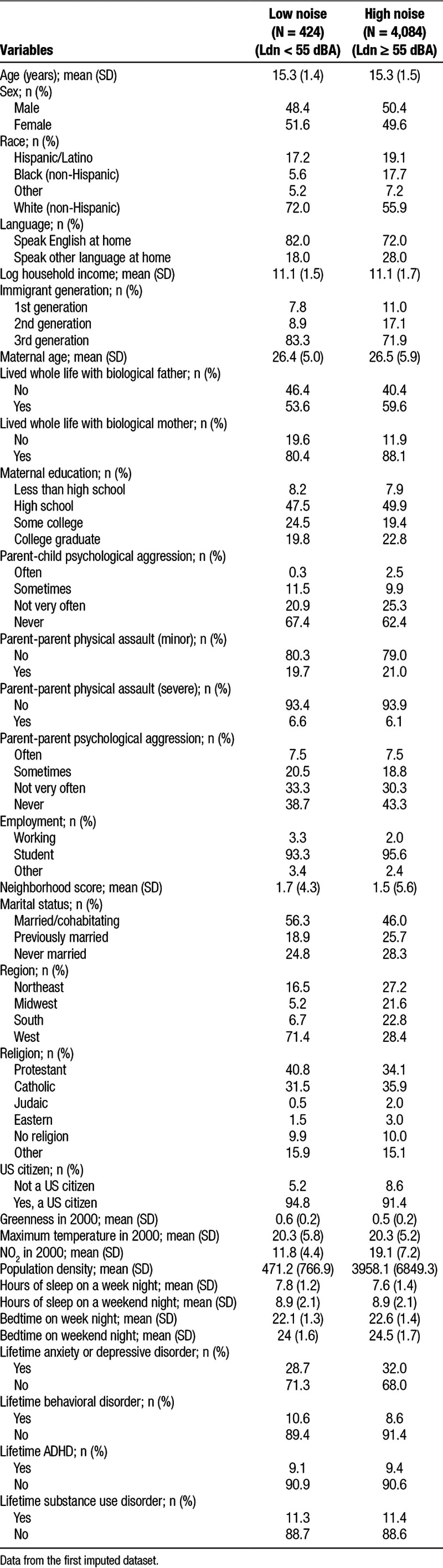
Demographic and community characteristics by noise level: NCS-A, United States, 2001–2004

Table 2 shows associations between living in a high- versus low-noise Census block group and sleep and mental health outcomes among urban adolescents, conditioning on numerous adolescent, household, and environmental covariates. Several of these covariates were potentially strong confounders: we observed moderate correlation between our exposure measure and NDVI (ρ = –0.50) and fairly strong correlation with NO_2_ (ρ = 0.75). High noise exposure above the US EPA threshold was associated with bedtimes that were about 30–40 minutes later on both weeknights and weekend nights (0.48 hours, 95% CI = –0.26, 1.23 and 0.65 hours, 95% CI = 0.32, 0.98, respectively), but not with total hours slept (–0.07, 95% CI = –0.83, 0.69 and 0.06, 95% CI = –0.55, 0.68, respectively, Table 2). These results were robust to sensitivity analyses in which we restricted to the area of support (Web Table 3; http://links.lww.com/EE/A50), treated noise as a continuous exposure (Web Table 4; http://links.lww.com/EE/A50), and used a data-drive noise dichotomization (Web Table 6; http://links.lww.com/EE/A50).

**Table 2:**
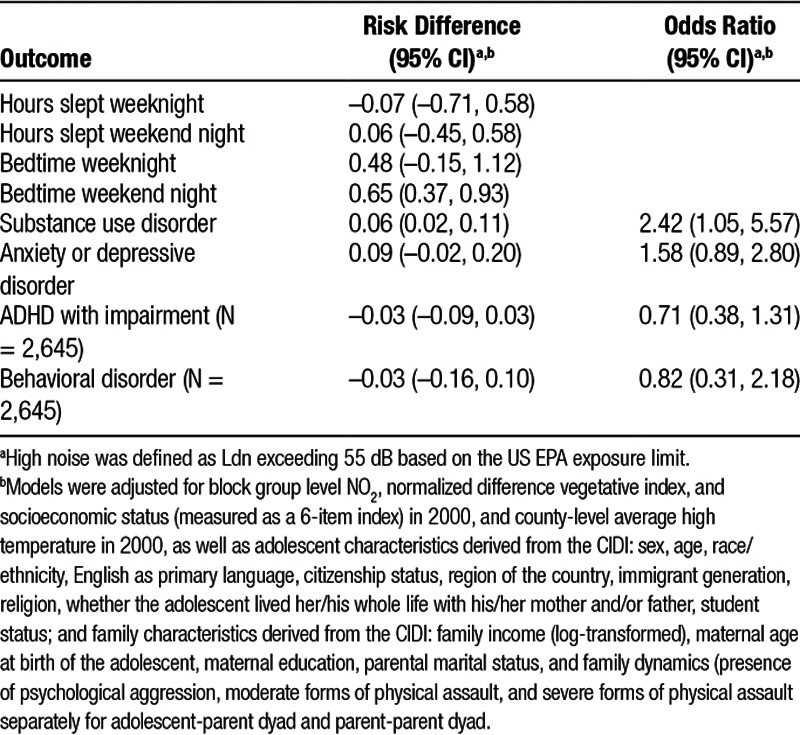
Associations between residence in an high- versus low-noise community and sleep and mental health among adolescents in the NCS-A, United States, 2001–2004 (N = 4,508)

In contrast to the robust results for later bedtimes, associations between high noise exposure and mental disorders among urban adolescents were mixed with wide CIs and less robust to sensitivity analyses. Living in a high- versus low-noise Census block group was associated with increased odds of anxiety or depressive disorder (Odds Ratio (OR): 1.58, 95% CI = 0.89, 2.80) and substance use disorder (2.42, 95% CI = 1.05, 5.57) but with reduced odds of behavioral disorders (0.82, 95% CI = 0.31, 2.18) and severe ADHD (0.71, 95% CI = 0.38, 1.31) (Table 2). Point estimates for anxiety or depressive disorder and substance use disorder were in similar directions using a higher threshold for noise dichotomization (Web Table 5; http://links.lww.com/EE/A50), using a data-driven noise dichotomization (Web Table 6; http://links.lww.com/EE/A50), and were similar for substance use disorder restricting to the area of support (Web Table 3; http://links.lww.com/EE/A50). However, CIs were generally too wide to be informative in these sensitivity analyses.

We repeated our primary analysis among adolescents in urban fringe and nonurban areas. Web Table 1; http://links.lww.com/EE/A50 gives descriptive statistics for adolescents living in these areas. Web Table 2; http://links.lww.com/EE/A50 shows that among these subgroups, all associations were null with wide CIs.

## Discussion

In a cross-sectional analysis of a nationally representative sample of US urban adolescents, we found that living in a community where average day-night noise exceeded the US EPA safety guideline of 55 dB was associated with approximately 30–40 minute later bedtimes. Associations with DSM-IV mental disorders were mixed, generally with wide CIs, and not robust across sensitivity analyses. Although we found that air pollution was highly correlated with noise, it could not explain the observed association between high noise levels and later bedtimes, consistent with prior studies.^56–58^ Our results were also not explained by area-level socioeconomic status, residential greenness,^59^ or compositional factors at the adolescent and household level. Recent European evidence suggests that noise harms human mental health and sleep,^1^ but, to our knowledge, this study represents the first analysis of noise and adolescent sleep and mental health in the United States. Further, by applying the nationwide noise model based on data collected 2000–2014 to 2010 US Census data, we estimated that over 70% of US adolescents lived in communities where day-night noise levels exceeded the US EPA exposure limit.

Early^60,61^ and more recent^62,63^ US studies have focused on transportation noise at schools, generally finding lower reading scores, cognition, and attention among students in louder learning environments. Most recent child and adolescent studies took place in Europe and reported positive associations between noise and cognitive impairment.^13,58,64^ Further studies among children and early adolescents have supported a relation between noise and hyperactivity/inattention symptoms^15,16,18–20,65^ and ADHD symptomology measured by DSM-IV criteria (although not diagnoses).^57^ The few studies that assessed the association between elevated noise and child/adolescent sleep found increased parent-reported sleep problems^3,14–16^ but not reduced sleep quality measured by wrist actigraphy.^14^ Almost no studies have included mid-late adolescents,^22,66^ as we did herein.

Noise activates the sympathetic nervous system, even during sleep,^1,2^ which can fragment sleep and reduce total sleep time. We found that adolescents living in high-noise areas had later bedtimes on both weeknights and weekend nights. We saw no difference in sleep duration, implying that high-noise exposure may shift sleep phase. Later bedtimes among adolescents have been associated with adverse mental health outcomes, substance use, and poorer academic performance.^67–71^ Similarly, negative health outcomes have been reported in association with greater differences in bedtime between weeknights and weekend nights.^71–74^ Health problems can result from sleep deprivation and circadian misalignment from later bedtimes.^75^

Although we found some evidence for a relation between high noise in urban areas and increased odds of substance use disorder and anxiety or depressive disorders, these results were not robust across sensitivity analyses. Prior studies have made similar conclusions, with a recent review describing evidence of the relation between noise and mental health in children as “heterogeneous and limited.”^76^ Several studies have reported associations between noise pollution and hyperactivity and inattention in children, including in a longitudinal context.^77^ However, we found no evidence for a relation between high-noise and odds of behavioral disorder and severe ADHD—even the directionality of point estimates differed across sensitivity analyses.

Contrasting our primary analytic results (Table 2) with the sensitivity analysis restricting to the area of support (Web Table 3 and Web Figure 2; http://links.lww.com/EE/A50) is instructive for interpreting these findings. Restricting to the area of support reduced the sample size by about 60% (from 4,508 urban adolescents to 1,880), indicating that residing in high-noise areas may be subject to significant structural confounding.^78^ For most urban adolescents, their levels of exposure to air pollution, greenspace, neighborhood disadvantage, etc., nearly perfectly predict whether or not they are exposed to noise levels greater than 55dB. Thus, by restricting to the area of support, we not only greatly reduced sample size and power, but we also effectively changed the estimated effect from the average treatment effect among urban adolescents to an effect that is less interpretable in that it applies to a subset of urban adolescents who have counterparts with similar propensity scores in the opposite exposure group. Point estimates differed between these two analyses, with those for ADHD and anxiety or depressive disorder demonstrating the largest differences, suggesting that that the relation between noise exposure and these disorders may be modified by important factors that differed between the representative sample and the subsample.

Contrasting our primary analytic results with the sensitivity analysis restricting to the area of support also demonstrated the extent of the challenge of estimating health effects of noise given the degree of structural confounding. We took several approaches to address confounding, including adjusting for numerous individual level, family level, and environmental covariates by full matching on the propensity score coupled with a doubly robust substitution estimator, as has been recommended previously.^79^ However, unobserved confounding likely remains.

The unexpected point estimates indicating reduced odds of ADHD and behavioral disorders in areas with high noise levels may reflect bias due to unobserved confounding, including measurement error. Prior work has found that the sensitivity and specificity of CIDI ADHD diagnoses may vary by characteristics such as race/ethnicity.^80^ ADHD and other behavioral disorder diagnoses stemming from caregiver or teacher reports also inherently include a degree of subjectivity and reliance on comparing the child/adolescent to his/her peers.^81,82^

Prior research suggests that noise sensitivity—internal states that increase the likelihood of noise annoyance^83^—may modify the relation between noise and health. In adults, noise sensitivity has predicted onset of depressive and psychological symptoms.^84^ In adolescents, higher morning saliva cortisol levels were correlated with high noise annoyance and living in high-noise areas.^85^ We lacked a measure of noise sensitivity or annoyance, and so could not assess its effect. Children, however, seem to report less annoyance than adults under the same noise conditions.^86,87^ Relatedly, psychological noise appraisal may also modify the relation between noise and health.^88^ Individuals may perceive noisy neighborhoods as lower quality. This in turn may constrain psychological restoration,^89^ limit physical activity,^90,91^ and reduce social contact.^92^

### Strengths and limitations

To our knowledge, only one prior study evaluated associations between noise and health nationwide in the United States^93^ and was among adults. Ours is the first to examine health effects among adolescents in the United States and one of the few to examine sleep and mental health outcomes among adolescents. We employed a well-documented, nationally representative sample of adolescents with information on both sleep patterns and DSM-IV mental health diagnoses. Many prior studies focused solely on aircraft^17,20,21,58,66,94^ or traffic-related noise.^3,14–16,18,22^ We implemented a nationwide sound model that incorporated diverse geospatial data to comprehensively account for the total burden of noise from anthropogenic sources.^37^ Another contribution was our analytic strategy. We employed propensity score matching coupled with a doubly robust estimator to improve control for potential confounding variables and a flexible modeling strategy. We categorized communities as high- and low-noise based on the policy- and health-relevant US EPA exposure limit.^29^ This has the advantages of being policy-relevant, making the stable unit treatment value assumption (SUTVA) more plausible,^95^ and facilitated our robust analytic strategy. However, it asks a research question about that particular US EPA threshold instead of estimating a more general relation between exposure to noise and adolescent sleep and mental health. Examination of other reasonable thresholds and a continuous relation with level of noise was the goal of several sensitivity analyses (Web Tables 3–6; http://links.lww.com/EE/A50). Comparing the results from the main analysis to these sensitivity analyses provides some evidence that there may be a general relation between increasing noise levels and later bedtimes but not for the other sleep outcomes or for mental health outcomes.

This study also had limitations. One central issue was mismeasurement/misclassification of the exposure. Measurement error was due to (at least) (1) model uncertainty; (2) aggregation uncertainty; and (3) misalignment of data years. In terms of model uncertainty, noise was predicted at 270 m resolutions from a model, the model-based prediction error is an area for future work. Aggregation uncertainty results from (1) having noise predictions in at 270 m resolution instead of a personal measurement of noise exposure (thereby ignoring locations beyond the residential block group where adolescents may have spent time or any noise abatement strategies employed in the home) and (2) from further aggregating the 270 m noise prediction to the block group level, resulting in standard errors of the sample mean. We have standard error estimates from the latter portion of aggregation error, from which we estimate 3% misclassification of the binary noise exposure using multiple overimputation.^96^ In addition, the noise model was built using data collected 2000–2014 to health data collected 2001–2004; the misalignment of years used also introduces some error, the form and extent of which are unknown.

In addition to measurement error, the type, quality, and annoyance of noise exposure were unmeasured (e.g., specific sources of noise like road or air traffic) but likely important factors in characterizing and identifying specific noise exposures that are important for adolescent sleep and mental health. The predictions from the noise model ignore such characteristics, potentially violating SUTVA.^95^ Although dichotomizing noise exposure at the US EPA limit makes SUTVA more plausible, the binary measure can nonetheless be meaningfully different for different subgroups in the NCS-A (e.g., due to different sources of noise). We stratified on level of urbanicity to minimize this issue, although we used crude urbanicity categorizations.

The study was cross-sectional, preventing us from establishing a temporal relation between exposure and outcome. It was also observational, likely resulting in residual confounding, as discussed above. Sleep patterns were measured by adolescent report; future studies may wish to use objective measures such as actigraphy to measure sleep patterns. Caregiver reports of adolescent behavioral symptoms were not available for all adolescents, which may have impacted our findings. Finally, studies have shown that the relation between noise and health may differ by sex,^3^ socioeconomic status,^4,97^ or baseline health status,^98^ but we lacked the sample size to assess relation by subgroup. Future studies should prioritize longitudinal designs, improved exposure assessment, and objective sleep measures, as well as assessment of whether certain subgroups of adolescents are more susceptible to the potential adverse effects of environmental noise.

## Conclusions

Nationwide, nearly 30 million adolescents lived in communities where Ldn exceeded the US EPA exposure limit in 2010. In a nationally representative sample of urban adolescents, we found that exceeding this limit was associated with later bedtimes. Interventions aimed at reducing exposure to environmental noise among adolescents may positively impact their sleep. Public health strategies to reduce noise exposure include direct regulation of sources of noise as well alterations to the built environment.^30^

## Conflicts of interest statement

The authors declare that they have no conflicts of interest with regard to the content of this report.

## Supplementary Material
