# Supplementary material for: Environmental noise and sleep and mental health outcomes in a nationally representative sample of urban US adolescents
Source: Environ Epidemiol. 2019 Jun 25;3(4):e056. doi: 10.1097/EE9.0000000000000056 (PMC6693982; doi:10.1097/EE9.0000000000000056)
Supplement: Supplementary file 1 [file ee9-3-e056-s001.pdf]

Supplementary Online Content for "Environmental noise and sleep and mental health outcomes in a nationally-representative sample of urban U.S. adolescents"

Contents

Web Appendix 1. Statistical approach

Web Figure 1. Covariate balance before and after matching among urban adolescents.

Web Appendix 2. Propensity score restricted analysis

Web Figure 2. Distribution of propensity scores by noise exposure groups among urban adolescents.

Web Table 1. Demographic and community characteristics by noise level among 1) suburban and 2) rural adolescents.

Web Table 2. Estimates and 95% confidence intervals (95% CI) of associations between noise and sleep and mental health outcomes among 1) suburban and 2) rural adolescents.

Web Table 3. Estimates and 95% confidence intervals (95% CI) of associations between noise and sleep and mental health outcomes, among urban adolescents, limited to the area of support.

Web Table 4. Estimates of average treatment effect of a 1-standard deviation increase in day-night average sound level on sleep and mental health outcomes among urban adolescents.

Web Table 5. Estimates and 95% confidence intervals (95% CI) of associations between noise and sleep and mental health outcomes not limiting to area of support, noise dichotomized at 65 dB, among urban adolescents.

Web Appendix 3. Hierarchical clustering analysis method

Web Table 6. Estimates and 95% confidence intervals (95% CI) of associations between noise and sleep and mental health outcomes not limited to area of support, noise dichotomized with hierarchical clustering, among urban adolescents.

References

This supplementary material has been provided by the authors to give readers additional information about their work.

Web Appendix 1. Statistical approach

Our analysis proceeded in several steps. We first imputed missing values using multiple imputation by chained equations, creating 30 imputed datasets (1). There was no missingness for our exposure of interest or for the mental health outcomes. Sleep outcomes were missing for 1% of the sample or less. Covariate missingness ranged from 0% (sex, age, race/ethnicity, region, language, and citizenship) to 43% (parental marital status). Doing an analysis ignoring the missingness assumes that the data are missing completely at random—that is, missingness is independent of both observed and unobserved variables (2). This is a strong and typically unrealistic assumption (2). Addressing missingness through multiple imputation instead assumes that the data are missing at random—that is, missingness can depend on observed variables that are included in the missingness model (2). We included all variables used in our analysis in all missingness models, which is necessary for congeniality (3), as well as many additional variables at the individual, family, and Census block group levels for a total of 122 variables and 40 additional second order interactions that improved model fit. We assessed the quality of the imputation by comparing densities of the imputed and observed values, as recommended (1). We completed analysis on each dataset and then pooled the results using Rubin's combining rules (4).

We then used full-matching on the propensity score (5) for living in a high-noise area to balance across the two exposure groups. **Web Figure 1** compares covariate balance before and after matching. All covariates were included in the propensity score model. Balance on sex was ensured by exact matching, and we tightly controlled for household income and neighborhood socioeconomic status by matching within calipers of those variables (0.2 standard deviations), given the importance of these factors in adolescent mental health (6, 7) Combining propensity score matching with more strict control for influential variables has been recommended previously (8). The resulting frequency weights from matching procedure were multiplied by the NCS-A sampling weights for analysis. We used a doubly robust substitution estimator, targeted minimum loss-based estimation (9), to estimate the effect of

exposure to noise levels above versus below the EPA limit of 55 dB for each outcome. This analytic approach offers several robustness advantages as compared to a standard regression approach: 1) it is doubly robust, meaning that one can misspecify either the treatment or outcome model without introducing bias; 2) because it is a substitution estimator, estimates are guaranteed to lie within the parameter space; and 3) the outcome model was data-adaptively fitted using an ensemble of machine learning algorithms (generalized linear models, Bayesian generalized linear models, multivariate adaptive regression splines, and generalized additive models), thereby further reducing reliance on correct parametric model specification (10) (propensity scores fit in the matching step described above were included for the treatment modeling portion of this estimator). We used the SuperLearner ensemble machine learning approach in model fitting, which weights the above specified algorithms to minimize 10-fold cross-validated prediction error. Research has found that combining full matching on the propensity score with targeted minimum loss-based estimation is a preferred analytic strategy for minimizing bias and variance (11).

Finally, all confidence intervals were adjusted to account for multiple testing using a false discovery rate of 5% (12).

Web Appendix 2. Propensity score restricted analysis method

We assessed the sensitivity of our results to extrapolations beyond the support of the data by limiting our analysis to the subsample (n=1880) of adolescents that have counterparts in the opposite exposure group with similar propensities to live in high-noise areas. Practically, this meant limiting the sample to adolescents whose propensity for living in high noise areas was less than the 99th percentile among those who actually lived in low noise areas and greater than the 1st percentile among those who actually lived in high noise areas (13–15). **Web Figure 2** shows this restriction. This approach is not compromised by practical violations of the positivity assumption (15), which would result in relying on extrapolation beyond the support of the data. However, it results in much smaller sample and loss of generalizability

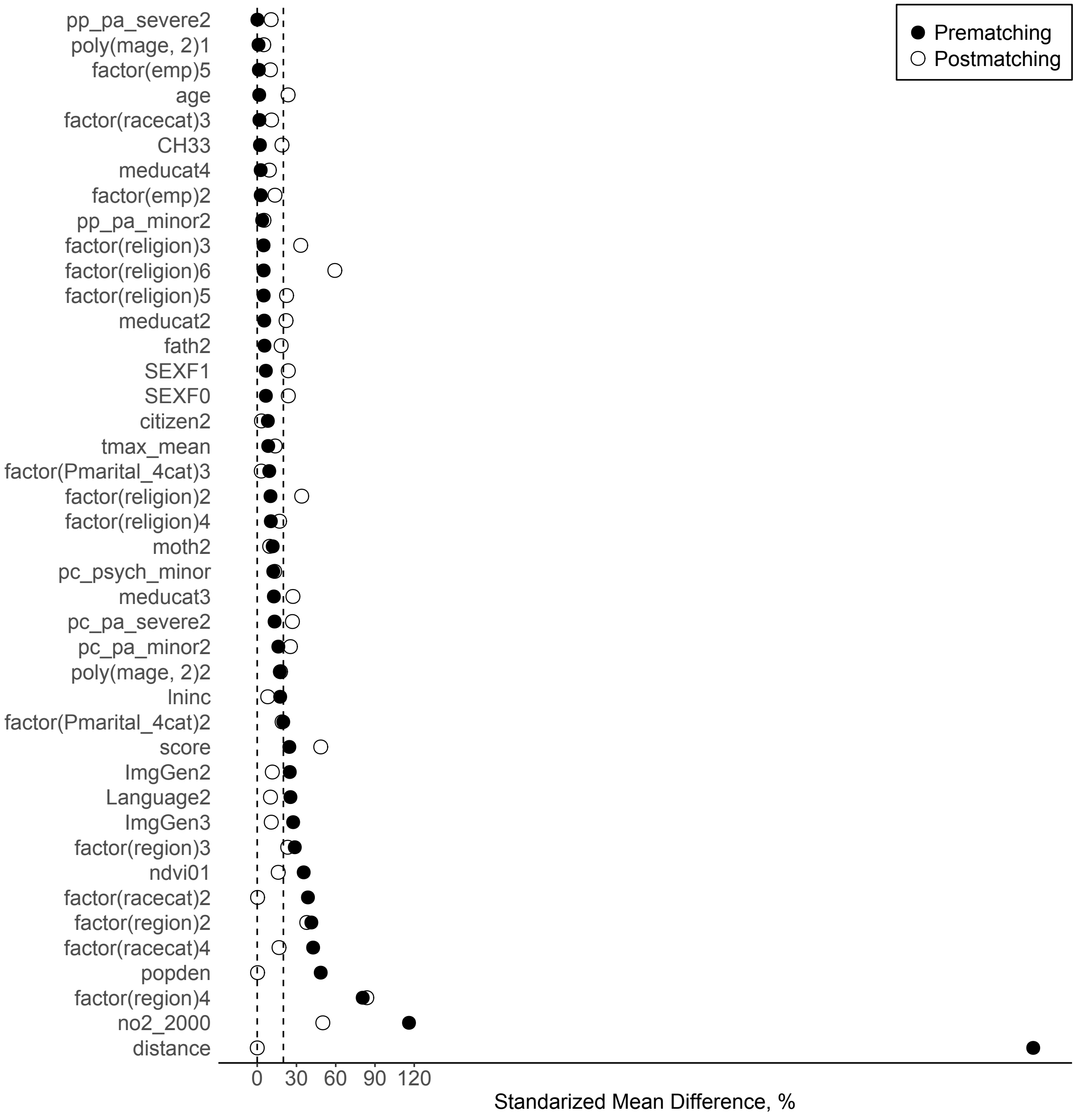

Web Figure 1: Covariate balance before and after matching.

of the results to the population of urban US adolescents.

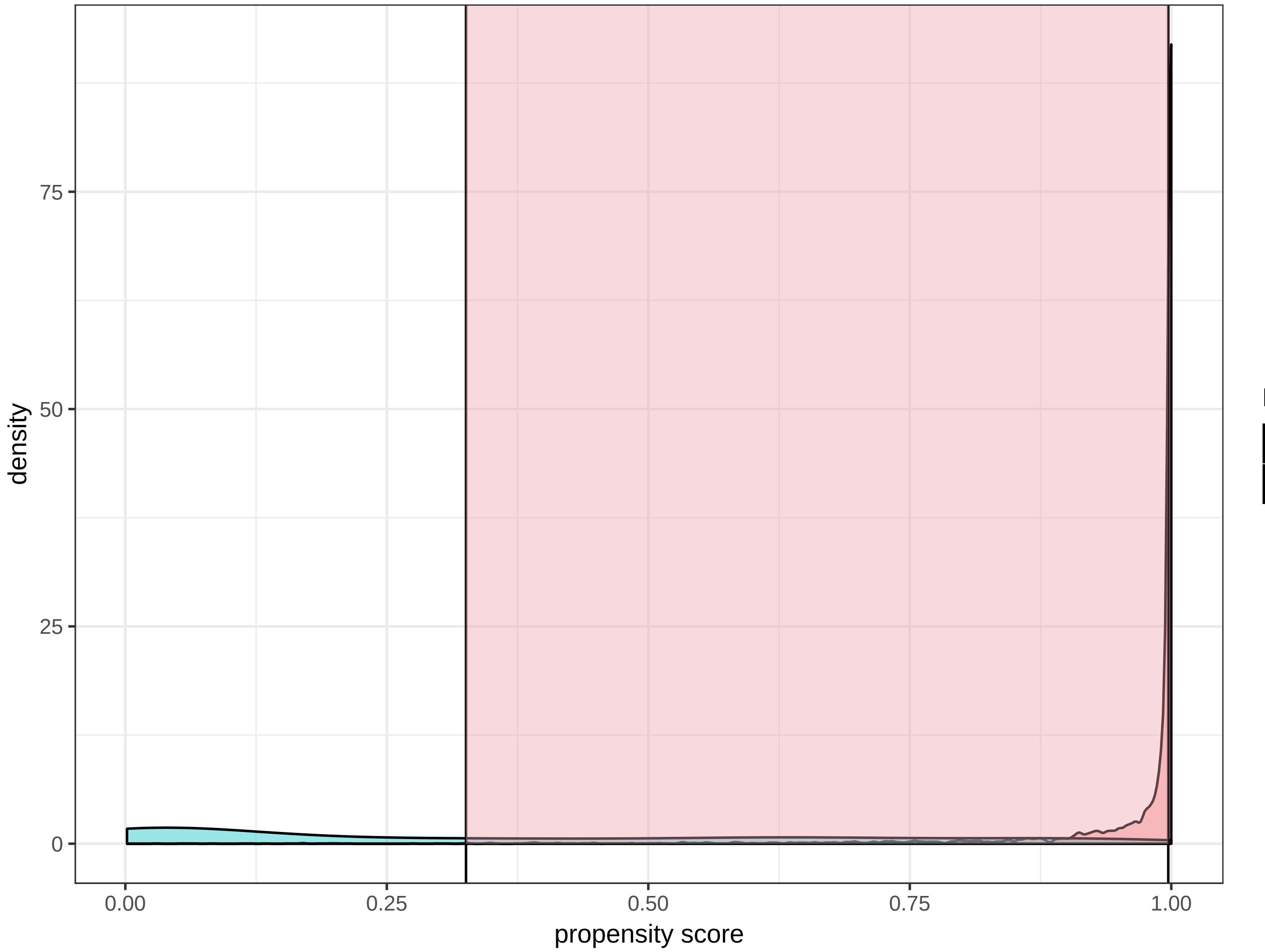

Web Figure 2: Distribution of propensity scores by noise exposure groups. Area of overlap used for sample restriction is highlighted in the pink rectangle. This area corresponds to N = 189 in the low-noise group (44.6% retained), and N = 1691 in the high-noise group (41.4% retained).

Web Table 1: Demographic and community characteristics by noise level among 1) suburban and 2) rural adolescents: National Comorbidity Survey Replication—Adolescent Supplement, United States, 2001–2004.

	Suburban (N = 3304)		Rural (N = 2311)	
Variables	Low Noise (N=838)	High Noise (N=2466)	Low Noise (N=1895)	High Noise (N=416)
Age (years), mean (SD)	15 (1.4)	15.2 (1.5)	15.1 (1.5)	15.0 (1.5)
Sex				
Male	53.4%	53.2%	49.2%	51.9%
Female	46.6%	46.8%	50.8%	48.1%
Race				
Hispanic/Latino	5.4%	15.2%	4.0%	8.7%
Black (non-Hispanic)	3.5%	17.3%	16.8%	7.0%
Other	2.8%	3.6%	2.6%	3.5%
White (non-Hispanic)	88.3%	63.9%	76.7%	80.9%
Language				
Speak English at home	89.1%	83.4%	91.7%	89.6%
Speak other language at home	10.9%	16.6%	8.3%	10.4%
Log household income, mean (SD)	11.2 (1.5)	11.0 (1.8)	10.7 (1.8)	10.9 (1.8)
Immigrant generation,				
1st generation	2.4%	4.1%	1.0%	3.0%
2nd generation	8.9%	8.6%	4.2%	5.1%
3rd generation	88.7%	87.4%	94.8%	91.9%
Maternal age, mean (SD)	27.2 (5.6)	26.1 (5.5)	25.0 (5.7)	25.6 (5.2)
Lived whole life with bio. father				
No	34.2%	43.8%	45.2%	42.6%
Yes	65.8%	56.2%	54.8%	57.4%
Lived whole life with bio. mother				
No	11.8%	11.3%	17.1%	10.1%
Yes	88.2%	88.7%	82.9%	89.9%
Maternal education				
Less than high school	6.1%	8.7%	9.3%	9.3%
High school	38.0%	47.4%	50.7%	47.7%
Some college	25.6%	22.4%	22.6%	21.7%
College graduate	30.3%	21.4%	17.3%	21.4%
Parent-child psych. aggr.				
Often	0.6%	1.5%	0.8%	0.5%
Sometimes	7.2%	8.7%	5.8%	6.4%
Not very often	25.1%	23.2%	20.3%	25.1%
Never	67.1%	66.5%	73.2%	68.0%
Parent-parent phys. asslt. (Minor)				

Variables	Low Noise (N=884)	High Noise (N=2254)	Low Noise (N=1987)	High Noise (N=440)
No	88.0%	80.4%	83.9%	78.5%
Yes	12.0%	19.6%	16.1%	21.5%
Parent-parent phys. asslt. (Severe)				
No	96.7%	92.8%	94.2%	94.3%
Yes	3.3%	7.2%	5.8%	5.7%
Parent-parent psych. aggr.				
Often	6.7%	8.6%	7.1%	8.5%
Sometimes	15.8%	18.6%	13.8%	19.6%
Not very often	32.6%	25.8%	26.0%	30.5%
Never	44.8%	46.9%	53.0%	41.4%
Employment, n (%)				
Working	1.6%	2.5%	2.7%	1.5%
Student	97.9%	95.0%	95.1%	96.5%
Other	0.5%	2.4%	2.2%	2.0%
Neighborhood score, mean (SD)	1.3 (3.9)	-1.2 (4.8)	-2.4 (2.9)	-2.2 (3.2)
Marital status				
Married/co-habitating	63.6%	50.0%	48.2%	40.6%
Previously Married	22.1%	30.4%	36.4%	45.5%
Never Married	14.3%	19.6%	15.4%	13.9%
Region				
Northeast	28.5%	6.6%	8.4%	0%
Midwest	21.3%	26.8%	23.3%	61.2%
South	27.9%	54.5%	59.2%	38.8%
West	22.4%	12.1%	9.0%	0%
Religion				
Protestant	42.0%	48.2%	68.6%	53.1%
Catholic	25.4%	25.6%	13.9%	22.0%
Judaic	1.3%	0.7%	0.2%	0.2%
Eastern	1.3%	0.5%	0.2%	0%
No religion	10.2%	10.0%	7.9%	10.0%
Other	19.8%	15.0%	9.2%	14.7%
US Citizen				
Not a US citizen	2.2%	3.6%	0.8%	3.1%
Yes, a US citizen	97.8%	96.4%	99.2%	96.9%
Greenness in 2000, mean (SD)	0.7 (0.1)	0.6 (0.1)	0.7 (0.1)	0.7 (0.2)
Max temperature in 2000, mean (SD)	18.3 (4.5)	21.2 (5.2)	19.7 (4.7)	18.4 (3.3)
NO_2 in 2000, mean (SD)	8.4 (2.3)	11.8 (4.7)	6.9 (2.2)	10.3 (2.7)
Population density, mean (SD)	415.4 (810.2)	1049.6 (1047.3)	168.7 (461.6)	824.8 (877.5)

Web Table 2: Estimates and 95% confidence intervals (95% CI) of associations between noise and sleep and mental health outcomes among 1) suburban and 2) rural adolescents in the National Comorbidity Survey Replication—Adolescent Supplement, United States, 2001–2004.

Outcome	Difference (95% CI)[a,b]	Odds ratio (95% CI)[a,b]
Suburban (N=3438)		
Hours slept weeknight	-0.44 (-1.25, 0.37)	
Hours slept weekend night	-0.04 (-0.65, 0.57)	
Bedtime weeknight	0.32 (-0.61, 1.25)	
Bedtime weekend night	0.27 (-0.06, 0.59)	
Substance use disorder	0.03 (-0.18, 0.24)	1.60 (0.08, 33.36)
Anxiety or depressive disorder	0.14 (-0.18, 0.45)	2.38 (0.31, 18.16)
ADHD with impairment	-0.15 (-0.54, 0.24)	0.45 (0.04, 4.49)
Behavioral disorder	-0.13 (-0.46, 0.21)	0.56 (0.12, 2.73)
Rural (N=2427)		
Hours slept weeknight	0.03 (-0.57, 0.64)	
Hours slept weekend night	0.12 (-0.99, 1.22)	
Bedtime weeknight	0.11 (-0.49, 0.71)	
Bedtime weekend night	0.08 (-0.83, 0.99)	
Substance use disorder	-0.01 (-0.23, 0.22)	0.96 (0.11, 8.08)
Anxiety or depressive disorder	-0.02 (-0.28, 0.23)	0.88 (0.24, 3.22)
ADHD with impairment	0.03 (-0.2, 0.25)	1.36 (0.11, 16.2)
Behavioral disorder	-0.07 (-0.33, 0.19)	0.57 (0.08, 4.07)

[a] High-noise was defined as day-night average sound levels exceeding 55 decibels based on the U.S. Environmental Protection Agency exposure limit.

[b] Models were adjusted for block-group level nitrogen dioxide, normalized difference vegetative index, and socioeconomic status (measured as a 6-item index) in 2000, and county-level average high temperature in 2000, as well as adolescent characteristics derived from the CIDI: sex, age, race/ethnicity, English as primary language, citizenship status, region of the country, immigrant generation, religion, whether the adolescent lived her/his whole life with his/her mother and/or father, student status; and family characteristics derived from the CIDI: family income (log-transformed), maternal age at birth of the adolescent, maternal education, parental marital status, and family dynamics (presence of psychological aggression, moderate forms of physical assault, and severe forms of physical assault separately for adolescent-parent dyad and parent-parent dyad.

Web Table 3: Estimates and 95% confidence intervals (95% CI) of associations between noise and sleep and mental health outcomes, among urban adolescents, limited to the area of support (N=1880).

Outcome	Difference (95% CI)[a,b]	OR (95% CI)[a,b]
Hours slept weeknight	-0.20 (-0.42, 0.03)	
Hours slept weekend night	0.49 (-0.15, 1.14)	
Bedtime weeknight	0.63 (0.46, 0.81)	
Bedtime weekend night	0.33 (0.04, 0.62)	
Substance use disorder	0.04 (-0.03, 0.1)	1.59 (0.64, 3.95)
Anxiety or depressive disorder	-0.17 (-0.34, 0.00)	0.49 (0.24, 1.00)
ADHD with impairment	0.03 (-0.04, 0.11)	1.79 (0.48, 6.6)
Behavioral disorder	-0.18 (-0.42, 0.06)	0.34 (0.09, 1.28)

[a] High-noise was defined as day-night average sound levels exceeding 55 dB based on the U.S. Environmental Protection Agency exposure limit.

[b] Models were adjusted for block-group level nitrogen dioxide, normalized difference vegetative index, and socioeconomic status (measured as a 6-item index) in 2000, and county-level average high temperature in 2000, as well as adolescent characteristics derived from the CIDI: sex, age, race/ethnicity, English as primary language, citizenship status, region of the country, immigrant generation, religion, whether the adolescent lived her/his whole life with his/her mother and/or father, student status; and family characteristics derived from the CIDI: family income (log-transformed), maternal age at birth of the adolescent, maternal education, parental marital status, and family dynamics (presence of psychological aggression, moderate forms of physical assault, and severe forms of physical assault separately for adolescent-parent dyad and parent-parent dyad.

Web Table 4: Estimates of average treatment effect of a 1-standard deviation increase in day-night average sound level on sleep and mental health outcomes among urban adolescents (N=4508).

Outcome	ATE[a,b]	95% CI
Hours slept weeknight	-0.006	-0.122, 0.111
Hours slept weekend night	-0.016	-0.172, 0.140
Bedtime weeknight	0.196	0.025, 0.368
Bedtime weekend night	0.048	-0.097, 0.192
Substance use disorder	-0.001	-0.022, 0.021
Anxiety or depressive disorder	0.025	-0.010, 0.060
ADHD with impairment	0.007	-0.032, 0.047
Behavioral disorder	0.013	-0.030, 0.055

Abbreviation: ATE, average treatment effect

[a] ATE for a 1-standard deviation (4.6 dB) increase in census block group noise level

[b] Models were adjusted for block-group level nitrogen dioxide, normalized difference vegetative index, and socioeconomic status (measured as a 6-item index) in 2000, and county-level average high temperature in 2000, as well as adolescent characteristics derived from the CIDI: sex, age, race/ethnicity, English as primary language, citizenship status, region of the country, immigrant generation, religion, whether the adolescent lived her/his whole life with his/her mother and/or father, student status; and family characteristics derived from the CIDI: family income (log-transformed), maternal age at birth of the adolescent, maternal education, parental marital status, and family dynamics (presence of psychological aggression, moderate forms of physical assault, and severe forms of physical assault separately for adolescent-parent dyad and parent-parent dyad.

Web Table 5: Estimates and 95% confidence intervals (95% CI) of associations between noise and sleep and mental health outcomes not limiting to area of support, noise dichotomized at 65 dB, among urban adolescents (N=4508).

Outcome	Difference (95% CI)[a,b]	OR (95% CI)[a,b]
Hours slept weeknight	-0.24 (-2.18, 1.71)	
Hours slept weekend night	-0.03 (-2.88, 2.83)	
Bedtime weeknight	0.24 (-1.59, 2.08)	
Bedtime weekend night	0.24 (-1.70, 2.19)	
Substance use disorder	0.07 (-0.27, 0.41)	1.82 (0.06, 57.81)
Anxiety or depressive disorder	0.07 (-0.65, 0.79)	1.37 (0.06, 30.8)
ADHD with impairment	0.07 (-0.31, 0.44)	1.77 (0.05, 66.8)
Behavioral disorder	0.08 (-0.4, 0.55)	1.60 (0.07, 35.59)

[a] High-noise was defined as day-night average sound levels exceeding 65 dB based on the U.S. Federal Aviation Administrations threshold. 9% (n=405) of the sample lived in communities where day-night average sound levels exceeded 65 dB.

[b] Models were adjusted for block-group level nitrogen dioxide, normalized difference vegetative index, and socioeconomic status (measured as a 6-item index) in 2000, and county-level average high temperature in 2000, as well as adolescent characteristics derived from the CIDI: sex, age, race/ethnicity, English as primary language, citizenship status, region of the country, immigrant generation, religion, whether the adolescent lived her/his whole life with his/her mother and/or father, student status; and family characteristics derived from the CIDI: family income (log-transformed), maternal age at birth of the adolescent, maternal education, parental marital status, and family dynamics (presence of psychological aggression, moderate forms of physical assault, and severe forms of physical assault separately for adolescent-parent dyad and parent-parent dyad.

Web Appendix 3. Heirarchical Clustering Analysis Method

The sensitivity of our results was again assessed using a data-driven method to dichotomize the data into high and low noise groups. We used hierarchical clustering, with the complete agglomeration method, to create two clusters with distances based on mean and maximum day-night average sound levels (DNL), daytime equivalent continuous sound levels, and nighttime equivalent continuous sound levels. This method allowed us to identify low noise and high noise groups from the joint empirical distributions of the data and to split the data into a group exposed higher noise levels and a group exposed to lower noise levels. Membership in the high-noise cluster was used as the exposure of interest and the analysis was carried out as described in Web Appendix 1.

Web Table 6: Estimates and 95% confidence intervals (95% CI) of associations between noise and sleep and mental health outcomes not limited to area of support, noise dichotomized with hierarchical clustering, among urban adolescents (N=4508).

Outcome	Difference (95% CI)[a,b]	OR (95% CI)[a,b]
Hours slept weeknight	-0.04 (-0.28, 0.21)	
Hours slept weekend night	-0.18 (-0.71, 0.36)	
Bedtime weeknight	0.25 (0.01, 0.48)	
Bedtime weekend night	0.20 (-0.14, 0.55)	
Substance use disorder	0.04 (-0.03, 0.11)	1.53 (0.66, 3.55)
Anxiety or depressive disorder	0.08 (-0.03, 0.19)	1.48 (0.85, 2.58)
ADHD with impairment	0.05 (0.00, 0.10)	2.73 (0.94, 7.89)
Behavioral disorder	0.00 (-0.15, 0.15)	1.05 (0.36, 3.05)

[a] Observations were split into a high and low noise level group using hierarchical clustering. High-noise was defined as membership in the cluster with higher average mean and maximum noise levels.

[b] Models were adjusted for block-group level nitrogen dioxide, normalized difference vegetative index, and socioeconomic status (measured as a 6-item index) in 2000, and county-level average high temperature in 2000, as well as adolescent characteristics derived from the CIDI: sex, age, race/ethnicity, English as primary language, citizenship status, region of the country, immigrant generation, religion, whether the adolescent lived her/his whole life with his/her mother and/or father, student status; and family characteristics derived from the CIDI: family income (log-transformed), maternal age at birth of the adolescent, maternal education, parental marital status, and family dynamics (presence of psychological aggression, moderate forms of physical assault, and severe forms of physical assault separately for adolescent-parent dyad and parent-parent dyad.

References

[1] Buuren Stef, Groothuis-Oudshoorn Karin. mice: Multivariate imputation by chained equations in R *J Stat Softw.* 2011;45.

[2] Stuart Elizabeth A, Azur Melissa, Frangakis Constantine, Leaf Philip. Multiple imputation with large data sets: a case study of the Children's Mental Health Initiative *American journal of epidemiology.* 2009;169:1133–1139.

[3] Meng Xiao-Li. Multiple-imputation inferences with uncongenial sources of input *Statistical Science.* 1994:538–558.

[4] Rubin Donald B. *Multiple Imputation for Nonresponse in Surveys*;81. John Wiley & Sons 2004.

[5] Stuart Elizabeth A, Green Kerry M. Using full matching to estimate causal effects in nonexperimental studies: examining the relationship between adolescent marijuana use and adult outcomes. *Dev Psychol.* 2008;44:395.

[6] Merikangas Kathleen Ries, He Jian-ping, Burstein Marcy, et al. Lifetime prevalence of mental disorders in US adolescents: results from the National Comorbidity Survey Replication–Adolescent Supplement (NCS-A) *Journal of the American Academy of Child & Adolescent Psychiatry.* 2010;49:980–989.

[7] Reiss F.. Socioeconomic inequalities and mental health problems in children and adolescents: A systematic review *Social Science and Medicine.* 2013;90:24-31.

[8] Rubin Donald B, Thomas Neal. Combining propensity score matching with additional adjustments for prognostic covariates *J Am Stat Assoc.* 2000;95:573–585.

[9] Laan Mark J, Rubin Daniel. Targeted maximum likelihood learning *Int J Biostat.* 2006;2.

[10] Laan Mark J, Polley Eric C, Hubbard Alan E. Super learner *Stat Appl Genet Mol Biol.* 2007;6.

[11] Colson K Ellicott, Rudolph Kara E, Zimmerman Scott C, et al. Optimizing matching and analysis combinations for estimating causal effects *Sci Rep.* 2016;6:23222.

[12] Benjamini Yoav, Hochberg Yosef. Controlling the false discovery rate: a practical and powerful approach to multiple testing *Journal of the royal statistical society. Series B (Methodological).* 1995:289–300.

[13] Stuart Elizabeth A. Matching methods for causal inference: A review and a look forward *Stat Sci.* 2010;25:1.

[14] Stürmer Til, Rothman Kenneth J, Avorn Jerry, Glynn Robert J. Treatment effects in the presence of unmeasured confounding: dealing with observations in the tails of the propensity score distribution—a simulation study *Am J Epidemiol.* 2010;172:843–854.

[15] Petersen Maya L, Porter Kristin E, Gruber Susan, Wang Yue, Laan Mark J. Diagnosing and responding to violations in the positivity assumption *Stat Methods Med Res.* 2012;21:31–54.
